# Pan-PPAR Modulation Effectively Protects APP/PS1 Mice from Amyloid Deposition and Cognitive Deficits

**DOI:** 10.1007/s12035-014-8743-4

**Published:** 2014-05-17

**Authors:** Markus P. Kummer, Rafael Schwarzenberger, Sakina Sayah-Jeanne, Mathieu Dubernet, Robert Walczak, Dean W. Hum, Stephanie Schwartz, Daisy Axt, Michael T. Heneka

**Affiliations:** 1 Clinical Neuroscience Unit, Department of Neurology, University of Bonn, Sigmund-Freud-Strasse 25, 53127 Bonn, Germany; 2 Genfit, Parc Eurasante, 885 av. Eugène Avinée, 59120 Loos, France; 3 German Center for Neurodegenerative Diseases, Ludwig-Erhard-Allee 2, 53175 Bonn, Germany

**Keywords:** Alzheimer, PPAR, Inflammation, Amyloid, Behavior

## Abstract

Alzheimer’s disease (AD) is a neurodegenerative condition that leads to neuronal death and memory dysfunction. In the past, specific peroxisome proliferator-activated receptor (PPAR)γ-agonists, such as pioglitazone, have been tested with limited success to improve AD pathology. Here, we investigated the therapeutic efficacy of GFT1803, a novel potent PPAR agonist that activates all the three PPAR isoforms (α/δ/γ) in the APP/PS1 mouse model in comparison to the selective PPARγ-agonist pioglitazone. Both compounds showed similar brain/plasma partitioning ratios, although whole body and brain exposure to GFT1803 was significantly lower as compared to pioglitazone, at doses used in this study. Oral treatment of APP/PS1 mice with GFT1803 decreased microglial activation, amyloid β (Aβ) plaque area, Aβ levels in sodium dodecyl sulfate- and formic acid-soluble fractions in a concentration-dependent manner. With a single exception of Aβ38 and Aβ40 levels, measured by ELISA, these effects were not observed in mice treated with pioglitazone. Both ligands decreased glial fibrillary acidic protein (GFAP) expression to similar extent and did not affect ApoE expression. Finally, GFT1803 increased insulin-degrading enzyme expression. Analysis of spatial memory formation in the Morris water maze demonstrated that both compounds were able to partially revert the phenotype of APP/PS1 mice in comparison to wild-type mice with GFT1803 being most effective. As compared to pioglitazone, GFT1803 (pan-PPAR agonist) produced both quantitatively superior and qualitatively different therapeutic effects with respect to amyloid plaque burden, insoluble Aβ content, and neuroinflammation at significantly lower whole body and brain exposure rates.

## Introduction

Alzheimer’s disease is clinically characterized by progressive memory loss and decline of cognitive functions. Besides the classical histopathological hallmarks, extracellular amyloid β (Aβ) deposition and neurofibrillary tangles of tau protein, neuroinflammation has been established as a major component [1]. Aβ derives from a larger precursor protein (APP) by subsequent cleavages by two aspartyl proteases, [beta]-site APP cleaving enzyme 1 (BACE1) and γ-secretase, resulting in the secretion of Aβ into the brain parenchyma [1]. Deposition of Aβ has been suggested to initiate the pathological sequalae which ultimately leads to the development of Alzheimer’s disease (AD) [2]. Additionally, oligomeric, soluble Aβ causes cellular toxicity and interferes with memory formation at the synapse thereby causing the observed memory loss [3, 4]. Therefore, strategies that interfere with the formation and deposition of Aβ or accelerate its removal hold therapeutic promise.

Although there is currently no curative treatment for AD patients, a number of epidemiological studies demonstrated that a sustained intake of several non-steroidal anti-inflammatory drugs (NSAIDs) reduces AD risk by as much as 80 % and delays its onset [5]. NSAIDs are mostly known as cyclooxygenase inhibitors, preventing prostaglandin and thromboxane synthesis from arachidonic acid, but additional cyclooxygenase-unrelated activities, such as binding to prostaglandin receptors, modulation of γ-secretase activity and activation of the peroxisomal proliferator receptor γ (PPARγ), a member of the nuclear hormone receptor superfamily, seem to be involved in AD prevention. Regarding the latter, it was rapidly suggested that protective effects arise from the ability of NSAIDs to ligate PPARγ and to inhibit neuroinflammation in the AD brain [6]. Of note, among different NSAIDs, indomethacin and ibuprofen were characterized as PPARγ agonists with anti-inflammatory properties [7]. PPARγ is expressed in cells of the myeloid lineage including macrophages and microglia and its activation inhibits nuclear factor kappa-light-chain-enhancer (NFκB), AP-1, and STAT-1-dependent signal transduction pathways, thereby suppressing key inflammatory cytokines, such as TNF-α, IL-1β, and Il-6 [8, 9].

The development of more specific ligands of PPARγ, namely the thiazolidinedione (TZD) class of oral antidiabetics has prompted experimental studies to further validate PPARγ-mediated neuroprotection in models of cerebral ischemia, Parkinson’s disease (PD), amyotrophic lateral sclerosis, and AD [10]. A preventive effect of the TZD pioglitazone on sodium dodecylsulfate (SDS)-soluble Aβ40 accumulation [11] as well as on micro- and astroglial activation and Aβ plaque burden [12] was shown in two different murine AD models, Tg2576 mice and APPV717I mice, respectively. Besides the Aβ-lowering effect, PPAR agonists have also been shown to suppress Aβ-mediated activation of microglia and prevented cortical or hippocampal neuronal cell death in vitro [13–15]. Mechanistically, PPARγ has been shown to affect Aβ metabolism by suppressing the immunostimulated BACE1 promoter via a PPAR response element [16, 17] thereby lowering BACE1 protein levels and, as a consequence, Aβ production.

Due to a number of side effects and adverse clinical observations, TZDs of the pioglitazone generation recently came under scrutiny, followed by the development of a further class of PPAR agonists, which activate not only PPARγ but also the two other isoforms, PPARα and PPARδ. The use of those pan-PPAR agonists is appealing since it has been reported that, in contrast to PPARγ, the expression of PPARα and PPARδ is reduced in AD brains [18]. PPARα function insufficiency may predispose neural tissue and cerebral microvasculature to exacerbated oxidative stress, since PPARα activation by fenofibrate and other agonists attenuates vascular damage through inhibition of lipotoxicity, inflammation, and reactive oxygen species [19]. Likewise, PPARδ was examined for possible neuroprotective effects. Using synthetic PPARδ-specific agonists neuroprotective effects have been observed in animal models of cerebral ischemia, multiple sclerosis, AD, PD, radiation-induced brain injury, and spinal cord injury [20–24].

In this study, we investigated the efficacy of GFT1803, a novel, experimental pan-PPAR agonist on amyloid burden, spatial memory formation, and neuroinflammation in the APP/PS1 mouse model.

## Material and Methods

### Animals

Age-matched, female APP/PS1 transgenic animals (# 005864, The Jackson Laboratory) [25] were on C57/Bl6 genetic background. Mice were mice fed on a normal mouse chow or on chow containing either 1 or 10 mg/kg GFT1803 or 50 mg/kg pioglitazone from 4 to 6 months of age. Mice numbers were *n* = 12 for 1 and 10 mg/kg GFT1803, *n* = 11 for APP/PS1, and *n* = 10 for 50 mg/kg pioglitazone. For pharmacokinetic studies, Swiss mice (Elevage Janvier, France) were used. Mice were housed under standard conditions at 22 °C and a 12-h light–dark cycle with free access to food and water. Animal care and handling was performed according to the declaration of Helsinki and approved by the local ethical committees.

### Gal4-PPAR Activation Assays

COS-7 cells were maintained in standard culture conditions (Dulbecco’s modified Eagle's minimal medium (DMEM)) supplemented with 10 % fetal calf serum, 1 % sodium pyruvate, 1 % essential amino acids, and 1 % streptomycin/penicillin at 37 °C in a humidified atmosphere of 5 % CO_2_. All tested compounds were dissolved in DMSO (Sigma-Aldrich). Cells were transfected using 2 μl JetPEITM (Polyplus transfection)/μg of DNA. Briefly, 40 μg of DNA was transfected in a 225-cm^2^ culture flask of adherent COS-7 cells (respecting the 1:50 ratio between the Gal4(RE)_TkpGL3 plasmid and the plasmid coding the nuclear receptor of interest (pGal4-hPPARα, pGal4-hPPARγ, pGal4-hPPARδ, pGal4-mPPARα, pGal4-mPPARγ, and pGal4-mPPARδ) or of the pGal4phi plasmid (negative control). Cells were seeded in 384-well plates at the density of 20,000 cells/well and then incubated for 4 h at 37 °C. The activation was performed by using the Genesis Freedom 200^™^ robotic unit (Tecan), in fresh medium supplemented with 2 % of synthetic serum, free of lipids (Ultroser^™^, Biosepra) supplemented with the tested compounds (compound of interest or reference molecules) or vehicle (DMSO 0.1 %). Luciferase activity was measured using the Steady-Glo Luciferase Assay System (Promega, Madison, WI, USA). All transactivation experiments were performed at least two times. Activation curves were realized using SigmaPlot (version 7.0 from SPSS, IBM) software. SigmaPlot was also used to fit the standard curves and then determine the specific EC_50_ values, maximum effect versus reference molecules and Hill slope. The E_max_ effect of GFT1803 was calculated relative to the maximal induction (plateau) obtained with the corresponding reference compound. The reference compounds for PPARα, PPARγ, and PPARδ were fenofibrate (100 μM), rosiglitazone (10 μM), and GW501516 (1 μM), respectively.

### Compound Pharmacokinetics Study in Mice

The compound was administered to 5-week-old, male Swiss mice (Elevage Janvier, France) by the PO route in a solution of 0.5 % methyl cellulose (Sigma-Aldrich; M0262) and 0.3 % Polysorbate 80 (Tween 80; Sigma-Aldrich P8074). At the precise time point, blood samplings were done at the retro-orbital sinus. Blood samples were collected into tubes containing both lithium and heparin, centrifuged at 2,500 rpm at 4 °C, and plasma was collected. Individual plasma aliquots were frozen at −20 °C (±5 °C) and stored until analysis.

Animals were perfused with 7 ml cold saline; the brain tissue was collected and frozen at −20 °C. The molecular and daughter ions were selected for each molecule by direct infusion into the MS-MS system. According to the expected sensitivity, eight-point calibration standards (1, 5, 10, 50, 100, 500, 1,000, and 5,000 ng/ml) were run using standard conditions which consist to liquid chromatography–tandem mass spectrometry (LC-MS/MS) system with C18 column after precipitation of the plasma proteins with acetonitrile before the start of the analytical test. Calibration standards were performed in each matrix (plasma and brain). Prior to analysis, 100 μl of each plasma sample was mixed with 300 μl acetonitrile. Following protein precipitation, samples were vortex mixed for 30 s, centrifuged for 5 min at 15,000 rpm and the supernatant was removed. Analysis was performed using LC/MS/MS determination according to previous analytical test results. Brains were homogenized with a potter using water (1:1, (*w*/*w*)). Homogenate (100 μl) was mixed with 100 μl of acetonitrile. The mixture was vortexed for 30 s and centrifuged for 5 min at 15,000 rpm. Brain homogenate supernatants were directly measured by LC/MS/MS using a C18 Kromasil column and API4000 (Applied Biosystem) or Quattro (Waters) mass spectrometer.

### Brain Protein Extraction

Snap-frozen brain hemispheres were extracted as previously described [26]. In addition, the SDS-insoluble pellet was extracted with 70 % formic acid in water. Formic acid was removed using a speed vac (Eppendorf), and the resulting pellet was solubilized in 200 mM Tris–HCl, pH 7.5.

### Tissue Preparation

After competition of the behavioral testing, mice were anesthetized using isoflurane and transcardially perfused with 15 ml phosphate-buffered saline. The brains were removed from the skull. One hemisphere was immediately deep-frozen for biochemical analysis, and the other was fixed in 4 % paraformaldehyde.

### ELISA

Quantitative determination of Aβ was performed using an electrochemiluminescence ELISA for Aβ_1-38_, Aβ_1-40_, and Aβ_1-42_ (Mesoscale). Samples SDS and formic acid (FA) fractions were at east 20 times diluted in 1 % Tx-100, 25 mM Tris–HCl pH 7.5, 150 mM NaCl.

### Histology

Serial sagittal cryosections (40 μm, four sections per mouse) were immunostained using antiserum against glial fibrillary acidic protein (GFAP) (DAKO, 1:800), antibody IC16 [27] against human Aβ1-15 (1:400), and rabbit polyclonal antiserum against Iba1 (WAKO, 1:200). For that, sections were treated 15 min at 80 °C in 100 mM citrate buffer pH 5.5 and afterwards with 50 % methanol for 10 min. Sections were washed in phosphate-buffered saline (PBS), permeabilized using PBST (PBST, 0.1 % Tx-100), and blocked for 1 h in 20 % goat serum in PBST. Primary antibodies were added in 20 % goat serum on PBST for 18 h at 4 °C while gently rocking. Secondary antibodies were added in 20 % goat serum on PBST for 1.5 h at RT. Sections were mounted with ImmuMount (Thermo Scientific, Bonn, Germany) on Superfrost slides.

For methoxy-XO4 [28] staining, sections were rinsed in PBS, incubated in 10 μM methoxy-XO4 in 40 % ethanol/60 % H_2_O adjusted to pH 10 with 0.1 N NaOH for 10 min at RT, washed three times with water, incubated for 2 min in 0.2 % NaOH in 80 % ethanol, and washed three times with water. Sections were analyzed using a BX61 microscope equipped with a disk scanning unit to achieve confocality (Olympus, Hamburg, Germany). Image stacks were deconvoluted using Cell^P (Olympus, Hamburg, Germany). Quantitative assessment of plaque areas was done using the MBF-ImageJ 1.43m software bundle (NIH, Bethesda, MA, USA).

### Protein Blotting

Samples were separated by 4–12 % NuPAGE (Invitrogen) using MES or MOPS buffer and transferred to nitrocellulose membranes. APP, APP C-terminal fragments (CTF), and Aβ were detected using antibody 6E10 (Covance) and the C-terminal APP antibody 140 (CT15) [29], IDE using antibody PC730 (Calbiochem), GFAP using anti-GFAP antiserum Z0334 (Dako), ApoE using antibody sc-6384 (Sant Cruz), and tubulin using antibody E7 (Developmental Studies Hybridoma Bank). For dot blot analysis, 10 μl samples containing 25 μM peptide were mixed with 200 μl PBS and transferred to nitrocellulose membranes. Immunoreactivity was detected by enhanced chemiluminescence reaction (Millipore), and luminescence intensities were analyzed using Chemidoc XRS documentation system (Biorad).

### Open Field Behavior

The open field consisted of a 61 × 61 × 61 cm Perspex box with dark walls and a white floor and was dimly illuminated. The open field was virtually divided into corridors along the walls (10 cm wide), corners (10 × 10 cm), and a center (40 × 40 cm). Mice were put in the middle of the open field. The mice were tracked for 5 min on three subsequent days using Ethovision software (Noldus, Wageningen, The Netherlands).

### Morris Water Maze

The Morris water maze used consisted of a 61-cm high plastic circular basin (diameter 1 m) approximately half-filled with water, with an invisible platform of 10 × 10 cm just under water in the middle of one of four equal virtual segments (quadrants). A white curtain surrounded the basin. Three asymmetrically applied intra-maze cues were presented to the animals. The water was made opaque using white dispersion color. The mice were subjected to one training session per day for eight consecutive days. One training session consisted of four trials of 40 s each. Time between trials was 10 s. The starting position in each trial was quasi-random. If a mouse did not succeed, it was put onto the platform for 15 s. On day 9, mice were put in the basin in the absence of the platform and the time spend in the quadrants was determined (probe trial). Mice were tracked using Ethovision software (Noldus, Wageningen, The Netherlands).

### Data Analysis

Results were expressed as mean ±SEM of at least three experiments. For normally distributed samples, Student’s *t* test was used for single comparisons of means and one-way ANOVA for multiple comparisons of means combined with the Tukey post test to evaluate statistical significance. The levels for statistical significance were *p < 0.05, **p < 0.01, and ***p < 0.001. All evaluations were conducted using Prism 5.03 (Graphpad, San Diego, CA, USA).

## Results

### GFT1803 is a Potent and Equilibrated Pan-PPAR Compound with a Significant Brain Penetration

GFT1803 is a diphenyl propane derivative and was designed to activate all mouse and human PPAR isoforms (α, δ, and γ) with comparable affinities and with moderate efficacy, as compared to classical PPAR reference agonists, i.e., fenofibrate, rosiglitazone, and GW501516. As shown in Table 1, PPARα/δ/γ activation profile of GFT1803 is equilibrated in terms of EC_50_ values (10–70 nM). The maximal efficacy for each of PPAR isoform (E_max_) is in the range of 57 to 84 % of the E_max_ value of the corresponding reference compound.

**Table 1:** Transactivation assays in COS-7 cells

	Gal4-PPARα (LBD)		Gal4-PPARγ (LBD)		Gal4-PPARδ (LBD)	
	EC_50_ (μM)	TOP (% ref)	EC_50_ (μM)	TOP (% ref)	EC_50_ (μM)	TOP (% ref)
Murine	0.02	78	0.01	65	0.01	84
Human	0.03	57	0.06	61	0.07	84

Transactivation assays were performed in COS-7 cells. The E_max_ effect of GFT1803 is calculated relative to the maximal induction obtained with the corresponding reference compound in the same experiment. The reference compounds for PPARα, PPARγ, and PPARδ were fenofibrate (100 μM), rosiglitazone (10 μM), and GW501516 (1 μM), respectively

**Fig. 1:**
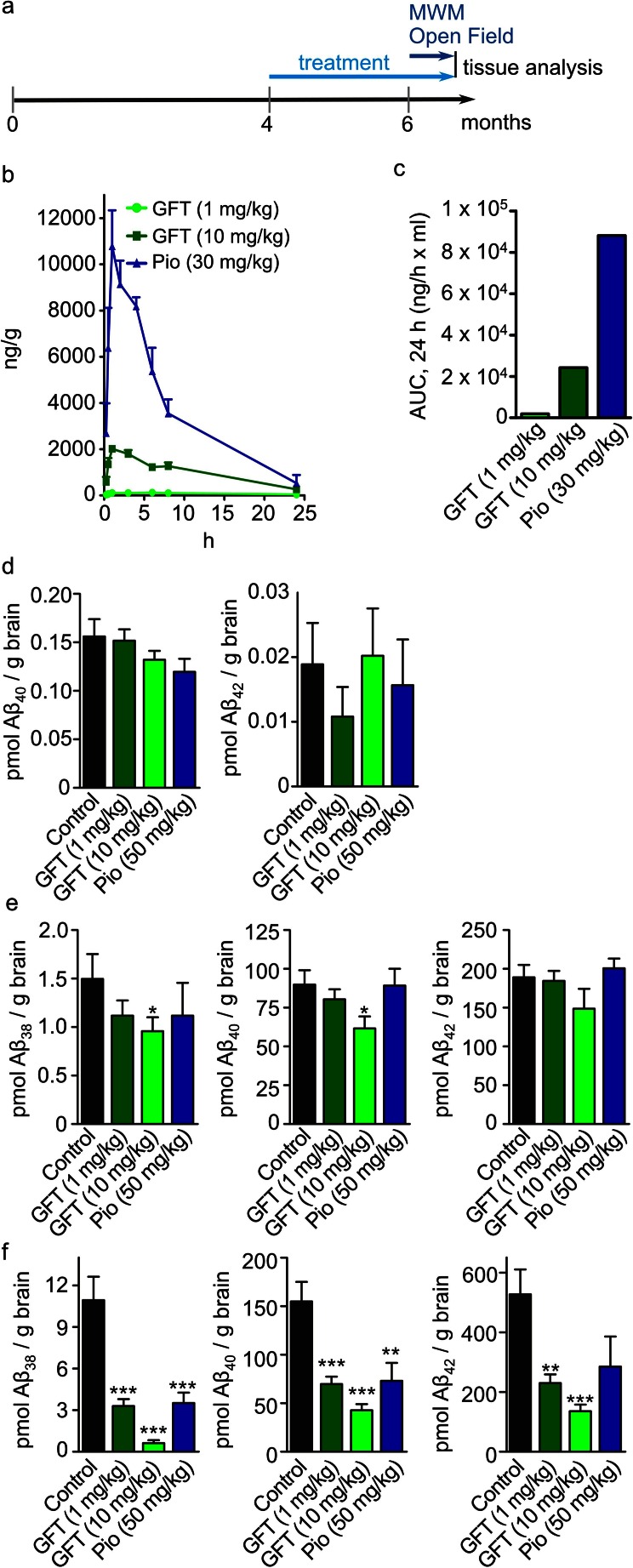
Experimental procedures, pharmacokinetics, and Aβ ELISA. **a** Scheme of the experimental procedures. Mice were treated from the age of 4–6 months with either GFT1803 or pioglitazone. At the beginning of month 6, mice were tested in the Morris water maze and in the open field paradigm for a total of 9 days. Drug treatment was continued during this time. Finally, mice were sacrificed and brains were subjected to histological analysis. **b** Pharmacokinetics of GFT1803 and pioglitazone in Swiss mice. Compounds were administered by oral gavage (GFT1803 at 1 and 10 mg/kg; pioglitazone at 30 mg/kg) and their brain concentrations were measured throughout 24 h (mean ±SD). **c** Determination of the area under the curve (*AUC*) values after 24 h of long exposure to the drugs. **d** Quantification of RIPA-soluble Aβ38, 40, and 42 from APP/PS1 mice treated with placebo (control), 1 mg/kg GFT1803, 10 mg/kg GFT1803, or 50 mg/kg pioglitazone (*pio*) by multiplex ELISA. Aβ38 could not be detected in this fraction. **e** Same as (**d**) but for SDS-soluble Aβ38, 40, and 42. **f** Same as (**d**) but for formic acid-soluble Aβ38, 40, and 42 (mean of *n* = 11 for control, *n* = 9 for pio, *n* = 12 for 1 mg/kg GFT1803, and *n* = 11 for 10 mg/kg GFT1803 ±SEM; one-way ANOVA, Tukey post hoc test; *p < 0.05, **p < 0.01, ***p < 0.001)

GFT1803 was also optimized to provide a significant central exposure upon oral administration. The central pharmacokinetic profile of GFT1803 at two different doses was established in the Swiss mice model and compared to that of pioglitazone (Fig. 1b, c). The central exposure of pioglitazone (30 mg/kg) was 3.57 times higher as compared to GFT1803 (10 mg/kg) and 50 times higher as compared to GFT1803 (1 mg/kg) (Fig. 1b, Table 2). However, both compounds showed similar brain/plasma partitioning ratio of 0.27–0.35 (Table 2).

**Table 2:** Values that correspond to the mean plasma and brain 24-h AUC (ng/h*ml)

	AUC [24 h] Brain (ng × h^−1^ × ml^−1^)	AUC [24 h] Plasma (ng × h^−1^ × ml^−1^)	Brain/plasma ratio
GFT1803 (1 mg/kg)	1,921	6,065	0.32
GFT1803 (10 mg/kg)	24,249	88,854	0.27
Pio (30 mg/kg)	88,112	252,551	0.35

In relative numbers, the central exposure to pioglitazone (30 mg/kg) was roughly 50 times higher as compared to GFT1803 (1 mg/kg) and roughly 3.57 times higher as compared to GFT1803 (10 mg/kg). Both compounds showed similar brain/plasma partitioning ratio of 0.27–0.35 (*AUC* area under the curve)

### GFT1803 Reduces Aβ Deposition and Neuroinflammation in APP/PS1 Mice

To test the efficacy of GFT1803 on amyloid pathology in an AD mouse model, we treated APP/PS1 mice for a period of 8 weeks, starting at the age of 4 months (Fig. 1a). GFT1803 and pioglitazone were both included in regular chow pellets to yield daily drug regimens of 1 mg/kg (GFT1803), 10 mg/kg (GFT1803), and 50 mg/kg (pioglitazone), with respect to an average daily food intake rate.

We differentiated the pools of Aβ in the forebrain by sequential extraction of brain homogenates starting with radioimmunoprecipitation assay buffer (RIPA), followed by 2 % SDS and finally by 70 % formic acid. Fractions were analyzed by multiplex ELISA for Aβ38, Aβ40, and Aβ42. We observed no effect of drug treatments on Aβ 40 and 42 species in the RIPA-soluble fractions (Fig. 1d). Aβ38 concentrations were below the detection limit. In the SDS-soluble fractions, treatment with GFT1803 (10 mg/kg) resulted in a modest but significant decrease of both Aβ38 and Aβ40 species (Fig. 1e). Pioglitazone showed no effect on Aβ in both RIPA and SDS-soluble fractions. Finally, the analysis of the most insoluble Aβ pool after formic acid extraction (Fig. 1f) revealed a strong and dose-dependent reduction for all three Aβ peptides in mice treated with GFT1803 (1 and 10 mg/kg). Treatment with pioglitazone (50 mg/kg) reduced both Aβ38 and Aβ40 in formic acid-soluble fractions with efficacy comparable to that of GFT1803 at 1 mg/kg, but had no statistically significant effect on Aβ42.

**Fig. 2:**
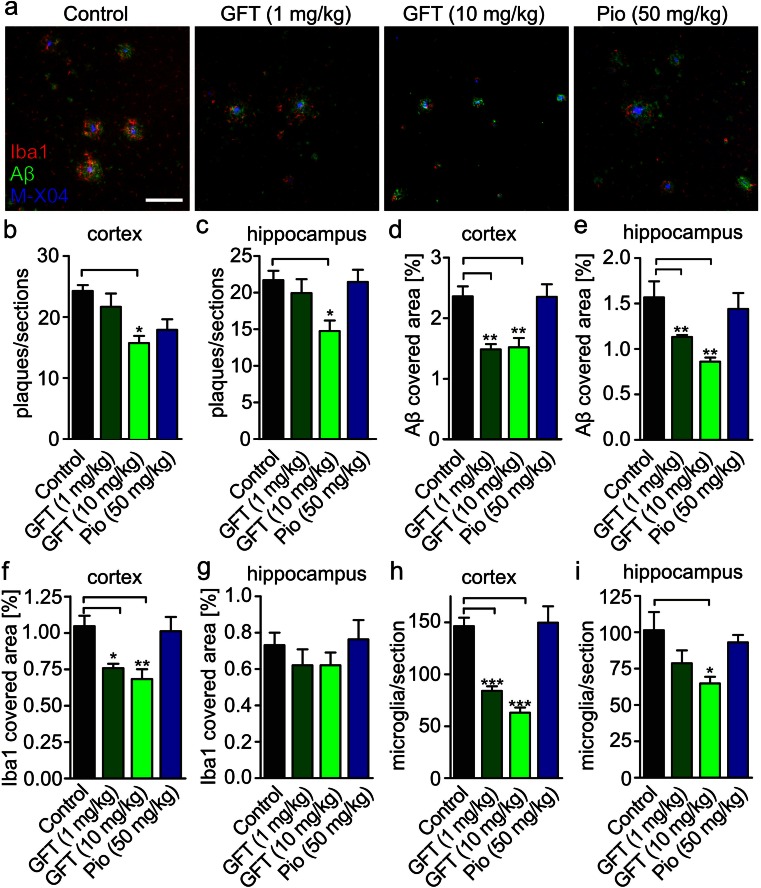
Histological analysis. **a** Brain section of APP/PS1 mice treated with placebo (control), 1 mg/kg GFT1803, 10 mg/kg GFT1803, or 50 mg/kg pioglitazone (*pio*) were stained with the amyloid dye methoxy-XO4 followed by immunostaining using antibodies IC16 against Aβ and the antibody anti-Iba1 to detect microglia. **b** Quantification of methoxy-XO4-positive plaques in the cortex and **c** in the hippocampus. **d** Determination of Aβ-covered area using antibody IC16 in the cortex and **e** in the hippocampus. **f** Determination of Iba1-covered area using antibody anti-Iba1 in the cortex and **g** in the hippocampus. **h** Quantification of Iba1-positive microglia in the cortex and **i** in the hippocampus (mean of *n* = 5 for control, *n* = 5 for pio, *n* = 6 for 1 mg/kg GFT1803, and *n* = 7 for 10 mg/kg GFT1803 ±SEM; one-way ANOVA, Tukey post hoc test; *p < 0.05, **p < 0.01, ***p < 0.001)

Further, we analyzed brain sections stained with the amyloid dye, methoxy-XO4 and by immunostaining for Aβ (IC16 antibody) and for the microglial marker Iba1. Aβ deposits were smaller and Iba1-immunoreactivity was decreased in sections from GFT1803 treated mice (Fig. 2a). Quantification of methoxy-XO4-positive plaques in samples from mice treated with GFT1803 (10 mg/kg) revealed a reduction of 48.5 % in the cortex and a reduction of 32 % in the hippocampus (Fig. 2b, c). However, no effect was observed for mice treated with either pioglitazone or with GFT1803 at 1 mg/kg. Determination of the Aβ-covered area by immunohistochemistry showed that GFT1803 (both doses) strongly reduced the Aβ load in the cortex (34 % for 1 mg/kg and 42 % for 10 mg/kg) and hippocampus (20 % for 1 mg/kg and 39 % for 10 mg/kg) (Fig. 2a, d, e). Anti-inflammatory activities of PPARs were extensively described in diverse systems, including the CNS. Therefore, we investigated microglial activation status in brain sections by using the activation marker Iba1. The quantification of the Iba1-covered area in the cortex showed a reduction of 43 % in mice treated with GFT1803 at 1 mg/kg and a reduction of 57 % in mice treated with GFT1803 at 10 mg/kg, but no effect for mice treated with pioglitazone (Fig. 2f, g). Similarly, the number of microglia per section was reduced in both cortex (42 % for 1 mg/kg and 57 % for 10 mg/kg) and to a lesser extent in hippocampus (38 % for 10 mg/kg), again with no effect in the pioglitazone treatment group (Fig. 2h, i).

**Fig. 3:**
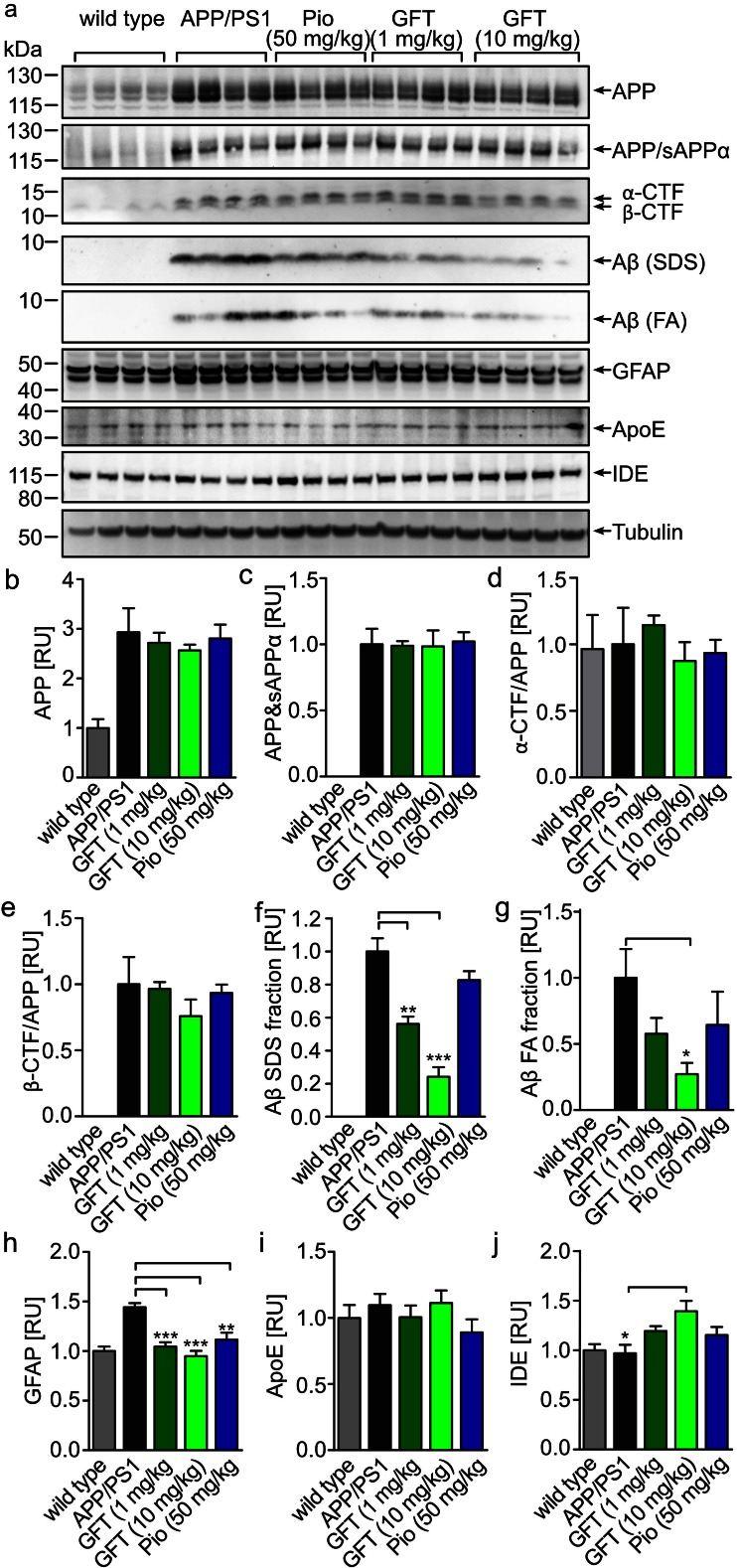
Biochemical analysis. **a** Immunoblot analysis of APP and APP-CTF in forebrain samples using antibody CT15, APP and Aβ using antibody 6E10, GFAP, IDE, ApoE, and tubulin. Densiometric quantification of **b** APP using CT15, **c** of APP/sAPPα using antibody 6E10, **d** α-CTF/APP using antibody CT15, **e** β-CTF/APP using antibody CT15, **f** Aβ in the SDS fraction, **g** Aβ in the FA fraction, **h** GFAP **i** of ApoE, **j** and of IDE (**b**–**g** are mean of *n* = 5 ±SEM, one-way ANOVA, Tukey post hoc test; *p < 0.05, **p < 0.01, ***p < 0.001)

Western blot analysis of RIPA-extracted forebrain homogenates revealed no changes in APP expression (Fig. 3a–c), nor in the α- and β-CTFs to APP ratio, suggesting unchanged APP processing (Fig. 3a, d, e). In contrary, we observed a reduction of Aβ in the FA and SDS fractions (Fig. 3a, f, g), which confirmed the ELISA results. The expression of the glial fibrillary acidic protein (GFAP), which is considered as an astrocytic activation marker and therefore indicator of glial inflammation, was found to be increased in APP/PS1 animals as compared to wild-type mice, whereas treatment with either GFT1803 or pioglitazone brought GFAP levels back to the level observed in wild-type mice (Fig. 3a, h). Since ApoE is implicated in Aβ removal [30, 31], we also tested if its expression was modulated in brains of treated mice, but ApoE expression remained stable (Fig. 3a, i). Interestingly, we observed that treatment with GFT1803 but not with pioglitazone induced the expression of the insulin-degrading enzyme (IDE), a key protease, able to degrade Aβ (Fig. 3a, j).

These data demonstrate that in APP/PS1 mice, the effect of GFT1803 on diverse AD pathology parameters was quantitatively and/or qualitatively superior as compared to pioglitazone. More specifically, GFT1803 performed better in reducing both highly insoluble (FA pool) and to a smaller extent moderately insoluble (SDS pool) Aβ peptides and also curbed microglial activation, which was not obtained by pioglitazone treatment at the applied dosage.

### GFT1803 Improves Spatial Learning and Memory in APP/PS1 Mice

**Fig. 4:**
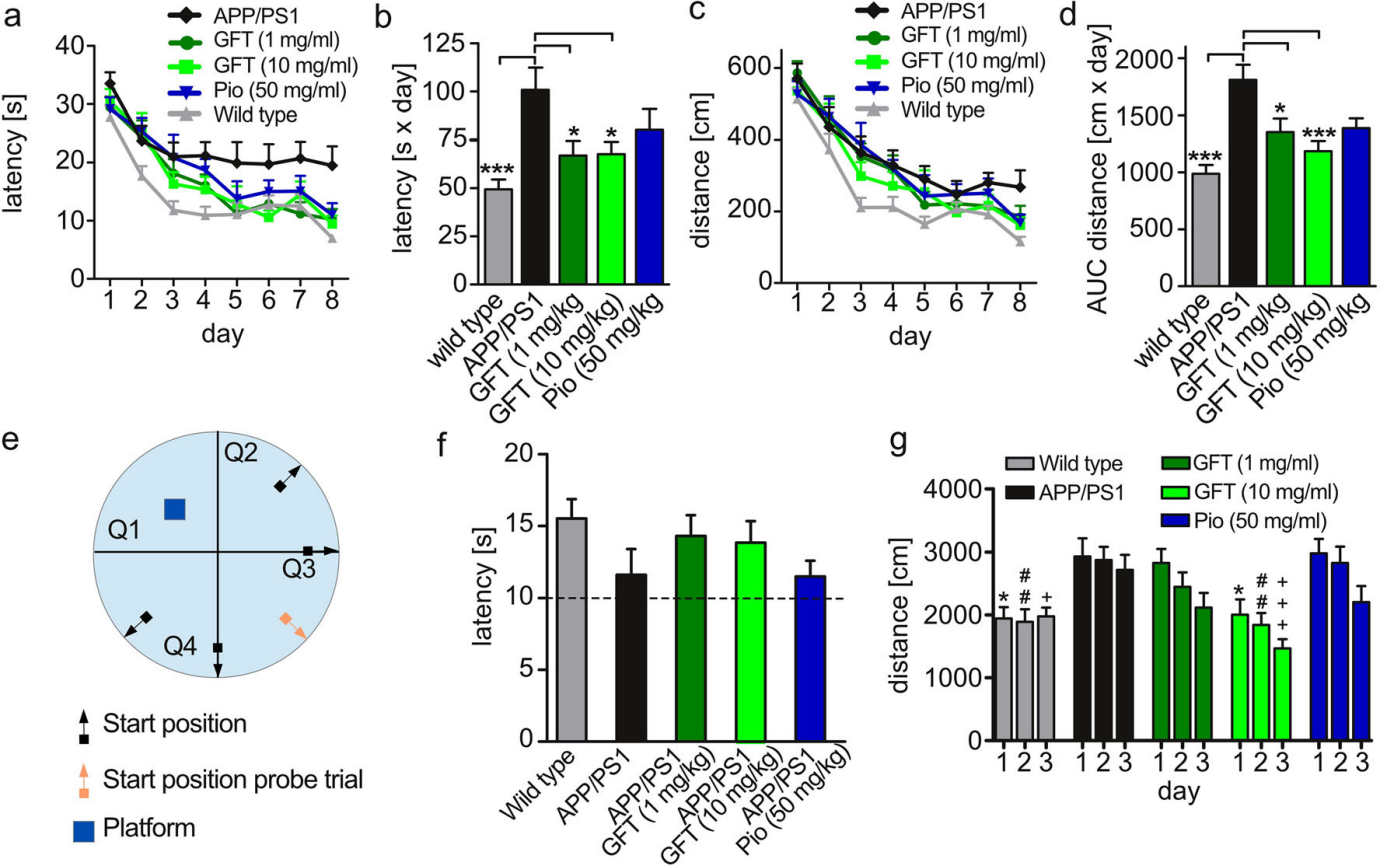
Behavioral analysis. Spatial memory learning of APP/PS1 mice treated with placebo (control), 1 mg/kg GFT1803, 10 mg/kg GFT1803, or 50 mg/kg pioglitazone (*pio*) in the Morris water maze. **a** Time needed to reach the hidden platform (latency in seconds). **b** Integrated time to reach the platform (area under the curve). **c** Distance traveled (in centimeters). **d** Integrated distance traveled (area under the curve). **e** Setup of the Morris water maze (*Q1–4* quadrant 1–4). **f** Mice were tested 1 day after the last trial day for 30 s in the absence of the platform. The time in the quadrants was measured and averaged for quadrants 2–4 (Q1-4 av.) (mean of *n* = 11 for control, *n* = 9 for pio, *n* = 12 for 1 mg/kg GFT1803, and *n* = 11 for 10 mg/kg GFT1803 ±SEM, Student’s *t* test, **p < 0.01, ***p < 0.001). **g** Distance traveled (in centimeters) in the open field assessment (mean of *n* = 11 for control, *n* = 9 for pio, *n* = 12 for 1 mg/kg GFT1803, and *n* = 11 for 10 mg/kg GFT1803 ±SEM; one-way ANOVA, Tukey post hoc test; *p < 0.05, **p < 0.01, ***p < 0.001)

We used the Morris water maze paradigm to evaluate the effect of GFT1803 treatment on hippocampal memory formation. As expected, wild-type mice performed best according to latency and distance, whereas APP/PS1 mice showed impairments for both parameters (204 and 183 % area under the curve (AUC) increase for latency and distance, respectively) (Fig. 4a–e). Treatment with both doses of GFT1803 reduced latency (25 % for 1 mg/kg and 34 % for 10 mg/kg vs. APP/PS1; AUC) as well as the distance swum (34 % for 1 mg/kg and 33 % for 10 mg/kg vs. APP/PS1; AUC). Pioglitazone in turn improved these parameters only in tendency. The probe trial demonstrated that none of the groups failed to form spatial memory (Fig. 4f). This indicates that GFT1803 improves spatial learning and/or memory retention in APP/PS1 mice.

Next, we assessed context-dependent habituation to a novel environment in the open field. APP/PS1 mice showed higher activity and less habituation than wild-type mice. Importantly, mice treated with 10 mg/kg GFT1803 habituated faster and more completely than APP/PS1 mice, suggesting improved contextual learning (Fig. 4g). We did not observe effects treating with the lower dose of GFT1803 or pioglitazone.

## Discussion

In this study, we provide a comprehensive analysis of the new pan-PPAR agonist GFT1803 for its impact on receptor activation, plaque burden, neuroinflammation, and spatial memory in a murine model of Alzheimer’s disease (AD). Previous studies have shown beneficial effects of PPARγ activation in several animal models of neurological disease [10], including cerebral ischemia [23], multiple sclerosis [32], Parkinson’s disease [33], and AD [12]. These experimental studies primarily used agonists of the thiazolidinedione class of antidiabetic drugs (TZDs), which were developed for the treatment of non-insulin dependent type 2 diabetes. Since TZDs have recently come under scrutiny for their potential cardiovascular side effects, some of these drugs are not longer considered as possible interventional therapeutics [34]. Positive reports of agonists activating other PPAR isoforms, which reduced Aβ plaque burden [22] and concomitant neuroinflammation [35] lead to the development of novel pan-PPAR ligands. Current characterization of these substances suggests they may demonstrate better benefit/risk ratios due to a balanced activation of all three PPAR isoforms, while at the same time keeping the positive properties reported for single receptor activation.

In the present study, preventive treatment of APP/PS1 mice with GFT1803 was initiated well before the onset of plaque deposition and strikingly suppressed Aβ deposition at later stages. Analyzing brain homogenates, the strongest reduction was observed in the extremely insoluble formic acid fraction of Aβ. This finding suggests that either the formation of Aβ deposits were efficiently prevented or the removal was specifically stimulated. The latter hypothesis is supported by recent findings showing that PPAR activation increased microglial clearance through CD36-dependent phagocytosis [36]. Given recent evidence that reduced Aβ clearance accounts for the majority of AD cases of sporadic nature [37], increasing microglial clearance through pan-PPAR modulation may offer a novel therapeutic strategy. Immunohistological analysis of the effect of pioglitazone on Aβ deposition revealed minor changes, whereas the ELISA results show a strong decrease in the FA fraction. This might be due to the fact that both methods do no describe the exact same pool of Aβ.

In AD, Aβ deposition and neuronal demise activate microglia through several surface molecules including toll-like receptors [38], which subsequently leads to the production and release of proinflammatory molecules including cytokines, chemokines, nitric oxide, and prostaglandins. This acute and sterile inflammatory action may initially serve the attempt to maintain cerebral homeostasis and increase clearance capacity of microglia. While usually this type of inflammation rapidly resolves upon pathogen removal, ongoing deposition of Aβ does not allow for resolution but leads to a chronic type of cerebral inflammation. Furthermore, a chronic proinflammatory environment is able to upregulate neuronal BACE1 thereby further fueling APP processing and Aβ generation [22, 39]. Likewise, inflammation-driven induction of NOS2 which goes along with massive production of nitric oxide induces the nitration-induced aggregation of Aβ in senile plaques [40].

Based on the overall detrimental action of chronic neuroinflammation, inhibitory interventions are sought. Preventive GFT1803 treatment of APP/PS1 mice reduced microglial immunoreactivity, suggesting suppression of the neuroinflammatory component in this model. Most likely, this anti-inflammatory action is due to activation of all three PPAR isoforms and not mediated by a single receptor, since all PPARs have been shown to exert anti-inflammatory action. Thus, PPARγ activation results in strong suppression of inflammation by transcriptional transrepression of NFκB [41]. This anti-inflammatory action of PPARγ ligands was shown to be neuroprotective in several experimental models of neuroimmunological and neurodegenerative disease [12, 23, 32, 33]. Likewise, the PPARα agonist fenofibrate protected neurons and axons against micro- and astroglia-derived nitric oxide-mediated toxicity in vitro and inhibited the secretion of the proinflammatory cytokines IL-1β, TNF-α, IL-6, and IL-12 p40 34, 35. Additionally, PPARα induces transcription of genes of the β- and ω-oxidation pathways that neutralize and degrade LTB4, a powerful chemotactic inflammatory eicosanoid, to regulate the inflammatory response [42]. Administration of GW0742, a potent PPARδ-selective agonist, significantly reduced amyloid plaque burden in the subiculum of 5xFAD mice, which was associated with decreased glial activation and increased expression of neprilysin, an amyloid-degrading, microglia-derived enzyme [22]. The antioxidant and anti-inflammatory actions of PPARδ agonists have been observed in a variety of cell types, including astrocytes and microglia. In particular, PPARδ can activate transcription of antioxidant genes, including catalase and superoxide dismutase [43, 44]. The improved learning and memory phenotype found in response to the treatment with GFT1803 is likely to arise of a combination of the above described Aβ-reducing and anti-inflammatory effects, as both, Aβ and inflammatory molecules have been shown to suppress memory relevant neurophysiological processes such as hippocampal long-term potentiation [45, 46]. In most of the parameters analyzed in this study, GFT1803 was superior when compared to treatment with the TZD pioglitazone at drug concentrations previously investigated in models of neurodegeneration.

Currently, the pioglitazone is tested in a phase III study to evaluate the efficacy of a long-term treatment with low-dose pioglitazone in delaying the onset of MCI in cognitively normal individuals at high risk. Even so, we did not detect strong effects in our mouse model, long-term, low-dose treatment with pioglitazone might still be effective.

Even so, we cannot rule out that the superior efficacy of GFT1803 was caused by yet non-described off-target effects, unrelated to the activation of PPARs; one might consider that targeting all three PPAR isoforms may represent a successful strategy to suppress cerebral amyloidosis and chronic inflammation. The data presented here identify GFT1803 as a highly efficient PPAR agonist, which prevents the development of pathological and behavioral hallmarks of AD in the AP/PS1 mouse model when given from early time points on. Further studies have to prove now whether these promising data hold true in human AD.

## Abbreviations

NFκB: Nuclear factor kappa-light-chain-enhancer
STAT: Signal transducer and activator of transcription-1
FA: Formic acid
SDS: Sodium dodecylsulfate
